# Emerging Marine Biotoxins in Seafood from European Coasts: Incidence and Analytical Challenges

**DOI:** 10.3390/foods8050149

**Published:** 2019-05-01

**Authors:** Pablo Estevez, David Castro, Ana Pequeño-Valtierra, Jorge Giraldez, Ana Gago-Martinez

**Affiliations:** 1 Department of Analytical and Food Chemistry, University of Vigo, Campus Universitario de Vigo, 36310 Vigo, Spain; paestevez@uvigo.es (P.E.); dcastro@uvigo.es (D.C.); apequeno@uvigo.es (A.P.-V.); jgiraldez@uvigo.es (J.G.); 2 EU Reference Laboratory for marine biotoxins, Campus Universitario de Vigo, 36310 Vigo, Spain

**Keywords:** climate change, emerging toxins, fish, mollusks, ciguatoxin, tetrodotoxin, N2a, LC-MS/MS

## Abstract

The presence of emerging contaminants in food and the sources of the contamination are relevant issues in food safety. The impact of climate change on these contaminations is a topic widely debated; however, the consequences of climate change for the food system is not as deeply studied as other human and animal health and welfare issues. Projections of climate change in Europe have been evaluated through the EU Commission, and the impact on the marine environment is considered a priority issue. Marine biotoxins are produced by toxic microalgae and are natural contaminants of the marine environment. They are considered to be an important contaminant that needs to be evaluated. Their source is affected by oceanographic and environmental conditions; water temperature, sunlight, salinity, competing microorganisms, nutrients, and wind and current directions affect the growth and proliferation of microalgae. Although climate change should not be the only reason for this increase and other factors such as eutrophication, tourism, fishery activities, etc. could be considered, the influence of climate change has been observed through increased growth of dinoflagellates in areas where they have not been previously detected. An example of this is the recent emergence of ciguatera fish poisoning toxins, typically found in tropical or subtropical areas from the Pacific and Caribbean and in certain areas of the Atlantic Sea such as the Canary Islands (Spain) and Madeira (Portugal). In addition, the recent findings of the presence of tetrodotoxins, typically found in certain areas of the Pacific, are emerging in the EU and contaminating not only the fish species where these toxins had been found before but also bivalve mollusks. The emergence of these marine biotoxins in the EU is a reason for concern in the EU, and for this reason, the risk evaluation and characterization of these toxins are considered a priority for the European Food Safety Authorities (EFSA), which also emphasize the search for occurrence data using reliable and efficient analytical methods.

## 1. Introduction

Marine biotoxins are natural contaminants of the marine environment, the incidence of which could be related to the proliferation of phytoplankton and associated with harmful algal blooms (HABs). The occurrence of HABs can also be linked to the presence of high nutrients (nitrogen, phosphorus), CO_2_ concentration, temperature, and specific climatic conditions. The ingestion of marine biotoxins can cause illness and even death to both aquatic organisms and humans [1]. Marine biotoxins accumulate in marine organisms, mainly in filter-feeding bivalve mollusks, and are becoming an important concern for public health.

Marine biotoxins can be classified depending on their solubility as hydrophilic and lipophilic. The hydrophilic toxins involve the regulated amnesic shellfish poisoning (ASP) and paralytic shellfish poisoning (PSP) and the emerging pufferfish poisoning (PFP). The regulated lipophilic toxins are associated with diarrhetic shellfish poisoning (DSP) and azaspiracid poisoning (AZP) whereas the unregulated ciguatera fish poisoning (CFP), cyclic imines (CIs), and neurotoxic shellfish poisoning (NSP) are considered emerging toxins. CFP toxins and CIs have been reported in fish and shellfish from EU waters while NSP toxins have not been reported [2,3,4,5]. Regulatory limits have been set for some groups of marine biotoxins according to European Union legislation in order to protect consumers and producers (Table 1). In spite of the increasing occurrence of HABs due to factors such as global ocean warming and anthropogenic changes, effective seafood monitoring programs have reduced the documented poisonings related to marine biotoxins and limit human exposure to contaminated products [6].

Climate change has profound implications for marine ecosystems [7] and non-indigenous species invasions are being recognized as an important element [8]. The impact on marine HABs is fraught with difficulties, but the range expansion of warm-water species and species-specific changes in the abundance and seasonal window of growth of HAB taxa is expected [9]. Regulated phycotoxins are monitored regularly with validated methodologies based on multianalyte methods, thereby decreasing human’s risk of intoxication [10,11]. On the other hand, emerging toxins are being reported, and new guidelines are needed about how to manage them.

As a consequence, the European Food Safety Authority (EFSA), at the request of the European Commission (EC) in 2006, has developed a series of scientific opinions concerning marine biotoxins. These opinions include groups currently regulated in the European Union (EU) legislation and emerging toxins that could be a future concern, as well as new global health risks due to the spread and prevalence in new geographical regions of groups already reported in the past as palytoxins (PTXs), ciguatoxins (CTXs), and tetrodotoxins (TTXs) [12,13,14].

Species of the genus *Ostreopsis* associated with PTXs were first reported in Hawaii and Japan but are currently distributed worldwide, and blooms have recently been reported in four European countries: France, Greece, Italy, and Spain [15].

Ciguatera fish poisoning (CFP) is endemic in certain tropical regions of the world but no indigenous cases had been described in Europe until the first occurrence in the Canary Islands (Spain) in 2004. Epidemiological surveillance activities reported nine outbreaks with a total number of 68 cases between 2008 and 2012. Therefore, CFP is an emergent risk in the Canary Islands with a persistent incidence and impact on public health [16]. In addition, recent reports of CTXs and primary causative species of the genus *Gambierdiscus* and *Fukuyoa* in regions of the Mediterranean Sea [17] and the Canary Islands [18] suggest a possible future concern about CFP in finfish originating from Europe.

The presence of tropical species such as *Gambierdiscus*, *Fukuyoa*, and *Ostreopsis* in temperate regions accords with suggestions of several researchers regarding climate change impact on the geographical expansion of tropical microalgae. This fact constitutes a serious threat to human health by ciguatera and PTXs intoxications in the future.

TTX has been associated with contaminations of pufferfish in Japan, but the incidence of TTX in bivalves from other geographical areas outside Europe has not been extensively evaluated; therefore, not much knowledge can be shared on this specific issue. The occurrence of TTX in bivalve mollusks was reported mainly in Japan [19] and New Zealand [20], although the recent emergence of tetrodotoxin in bivalve mollusks in different locations across Europe, such as UK [21], Greece [22], Netherlands [23], Italy [24], and Spain [25], has generated the necessity to explore other areas in Europe in order to investigate the presence of TTX. Furthermore, the Health Authorities in the EU Commission, in agreement with the Competent Authorities from the different EU Member States, requested advice from the EFSA regarding risk evaluation and characterization of TTX in bivalves from the EU. An EFSA opinion was published in March 2017 [14] in which the recommendation of the evaluation of more occurrence data on TTX and its analogs in edible parts of marine bivalves and gastropods from different EU waters was included. The occurrence data should be obtained using EU approved methods.

The spread and increase of HABs worldwide are affecting European coasts where new emerging toxins are being detected and, therefore, causing concern to the EU Health Authorities. As a consequence, the EFSA has been requested to evaluate the risk of the presence of different groups of marine biotoxins and particularly CTXs and TTXs since both are responsible for the contamination of fish and bivalves in different EU coastal areas.

The aim of this work is to show some results obtained in the characterization of the risk of CTX and TTX in seafood from EU coastal areas. To accomplish this aim, results obtained using different methodologies for the screening and confirmation of these toxins—in particular, neuroblastoma cell assay (N2a) and liquid chromatography coupled to tandem mass spectrometry, respectively—are presented and discussed.

## 2. Ciguatoxins (CTXs)

**Figure 1:**
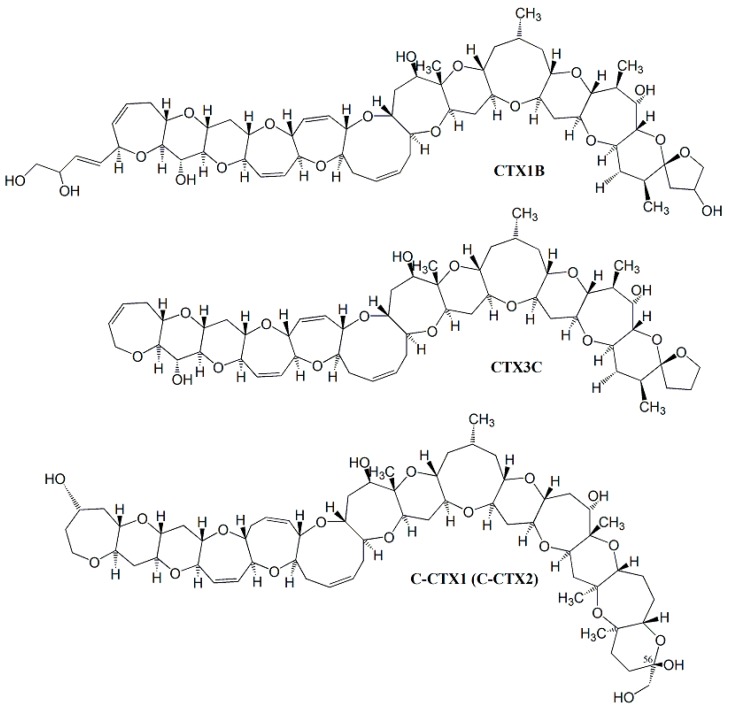
Chemical structures of the mayor ciguatoxins: CTX1B and CTX3C and C-CTX1 and its epimer C-CTX2 in C-56.

CTXs are lipophilic toxins produced by dinoflagellates of the genus *Gambierdiscus spp*. and *Fukuyoa spp*. [26,27]. These toxins are the cause of CFP following the ingestion of contaminated fish, and can lead to neurological, gastrointestinal, and cardiovascular disorders [28]. CTXs are found in the Pacific (P) and Indian (I) Oceans, the Caribbean Sea (C), and, more recently, in temperate waters of the Eastern Atlantic, mainly in the Canary Islands (Spain) and Madeira (Portugal), are are named P-CTXs, I-CTXs, and C-CTXs [2,29,30]. CTX1B and CTX3C are responsible for CFP in the Pacific and Indian Ocean [31,32], I-CTXs has not yet been elucidated [33], and C-CTX1 is the main CTX analog in the Caribbean Sea and seems to be the emerging analog in EU waters [34,35,36] (Figure 1).

The main limitation in CTX research is the lack of reference materials or standards [37]. This limitation together with the lower concentration of CTXs present in toxic fish makes advancements in the development of reliable analytical methods difficult. The US Food and Drug Administration (FDA) proposed a guidance level based on mouse bioassay results and applying a 10-fold threshold security factor, establishing 0.01 ng/g for the most toxic congener CTX1B and 0.1 ng/g for C-CTX1 [38,39].

There are different approaches to determining CTXs in fish samples. As with many other marine biotoxins, the mouse bioassay (MBA) was first used due to the above-mentioned unavailability of reference material. However, the lack of specificity of this assay as well as its other multiple drawbacks, including animal protection and welfare, has led to the development of alternative detection methods [40].

In the monitoring of CTXs, two different approaches are recognized that are considered complementary. First, samples are screened through highly sensitive methods such as the cytotoxicity assay neuroblastoma 2-a (N2a), receptor binding assay (RBA), or an immunochemical assay Enzyme-Linked ImmunoSorbent Assay (ELISA) [41,42,43]. Sample screening is followed by liquid chromatography coupled to low- or high-resolution mass spectrometry (LC-LRMS or LC-HRMS) analysis, which is the most widespread method used for CTX confirmation [44]. 

**Figure 2:**
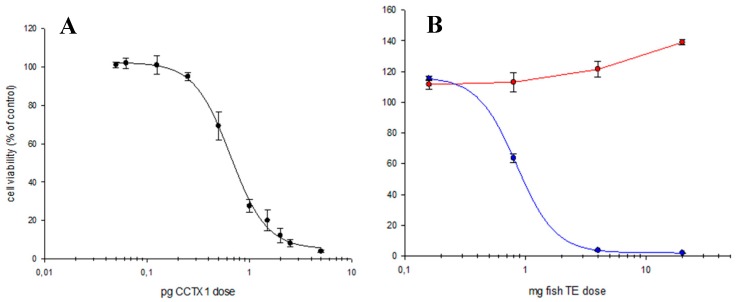
Example of N2a cytotoxicity results. (**A**) The C-CTX1 standard with the addition of ouabain/veratridine (O/V); (**B**) The amberjack (*Seriola fasciata*): red line, sample without the addition of O/V; blue line, toxic response in the sample with the addition of O/V. Data from [47]. N2a, neuroblastoma 2-a.

The most successful screening method seems to be N2a, which was initially described by Manger et al. [45]. This method allows the identification of a sample contaminated with CTX-like compounds with high sensitivity and evaluates the contribution of all the toxic analogs present in the sample. This method is based on the action mode of CTXs, which activates Na channels to induce cell death. The addition of ouabain (O) and veratridine (V) is required for this purpose, respectively blocking the Na^+^/K^+^ pump and the voltage-gated Na^+^ channel to increase the intracellular Na^+^ obtaining CTX-like specificity [46] (Figure 2). Immunochemical methods, such as the ELISA developed by Tsumuraya et al. [43], are rapid and sensitive but at present they are limited to a few P-CTXs.

Different LC-HRMS methods were recently developed for confirming CTXs based on their exact mass. These methods have the advantage of confirming the different CTXs without the availability of standards being necessary. On the other hand, sensitivity is not adequate and needs to be further optimized in order to detect levels around the guidance level [44,48,49]. Concerning LC-MS/MS (LRMS), two different approaches for CTXs confirmation are possible. The multiple reaction monitoring (MRM) of different water losses typical of cyclic polyether compounds is favored by the use of acetonitrile mobile phases [50]. On the other hand, MRM of the highly stable sodium adduct [M + Na]^+^ favors using methanol as a mobile phase [51]. The first approach has a lower sensitivity than the second and is more extended when the samples have a higher concentration of CTXs. The monitoring of [M + Na]^+^, despite having increased sensitivity, is limited by the availability of reference materials. It also requires adequate sample pretreatment due to the lack of specificity provided by the monitoring of a single [M + Na]^+^ and matrix interference can be easily misidentified with CTXs congeners.

Regarding P-CTXs, MBA for toxin evaluation followed by the method proposed by Yogi et al. [52] based on the above-mentioned monitoring of [M + Na]^+^ is the most extended approach. This approach allows the monitoring of up to 15 different CTXs and also gambierol and gambieric acids A and B, meeting the US FDA guidance level. A different approach was followed for C-CTX1 monitored in the Caribbean Sea. N2a was used to obtain a semi-quantitative value of the total toxicity whereas LC-MS/MS was used to further confirm C-CTX1 monitoring three water losses [53].

Different optimizations were done to improve the sensitivity, such as optimizing the electrospray ionization parameters through the design of experiments [54]. An LC-MS method was developed for maitotoxin (MTX) and CTXs monitoring in algal and fish samples [55].

**Figure 3:**
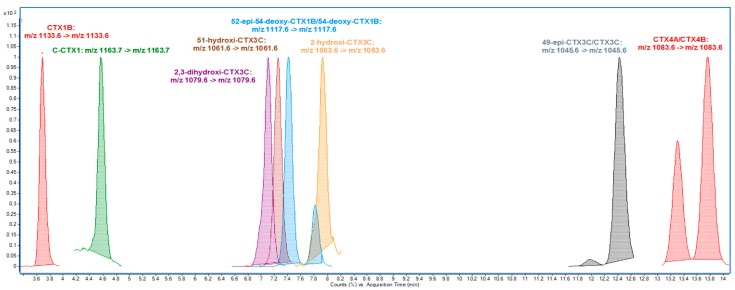
Example of LC-MS/MS (MRM) analysis monitoring the sodium adduct as a precursor and product ion in a qualitative mixture of P-CTXs and a C-CTX1 standard following the conditions described by [56]. Data from [47].

More recently, Estevez et al. developed a method for CTXs monitoring in EU waters in the absence of reference materials [56]. This method combines first identification and quantitation based on the monitoring of the sodium adduct [M + Na]^+^ of different P-CTXs and C-CTX1 according to the guidance levels established by US FDA (Figure 3). This is followed by a confirmatory analysis of the main toxin responsible for the contamination, C-CTX1. This confirmatory analysis allows unambiguous confirmation of C-CTX1 not only by monitoring water losses but also two specific fragments of the molecule formed at high collision energies, which are useful markers to detect new C-CTXs analogs.

**Figure 4:**
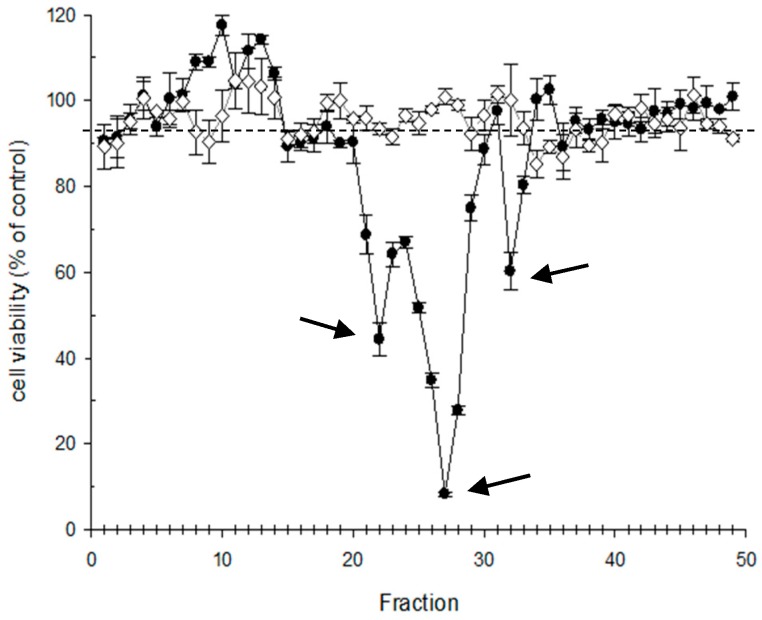
Example of N2a cytotoxicity profile of HPLC fractionated amberjack sample. Three prominent cytotoxic peaks can be observed corresponding to different C-CTXs analogs. White dots: sample without ouabain/veratridine treatment; black dots: sample with ouabain/veratridine treatment. Data from [47].

Further advancements in monitoring CTX in EU waters was done by [47] by developing a sample fractionation method coupled to N2a toxin evaluation of the different fractions to identify further the different C-CTXs analogs contributing to the total toxicity (Figure 4). Following this approach, C-CTX1 was detected and isolated as the main agent responsible for the toxicity and three putative C-CTXs were described by Pottier et al. in contaminated fish samples from the Caribbean Sea [57,58]. This approach also helped in the characterization of CTX profiles from Macaronesia, an emerging region in Europe, and gives evidence that the CTX profiles in this geographical region seem to be similar to those detected in the Caribbean Sea.

## 3. Tetrodotoxins (TTXs)

**Figure 5:**
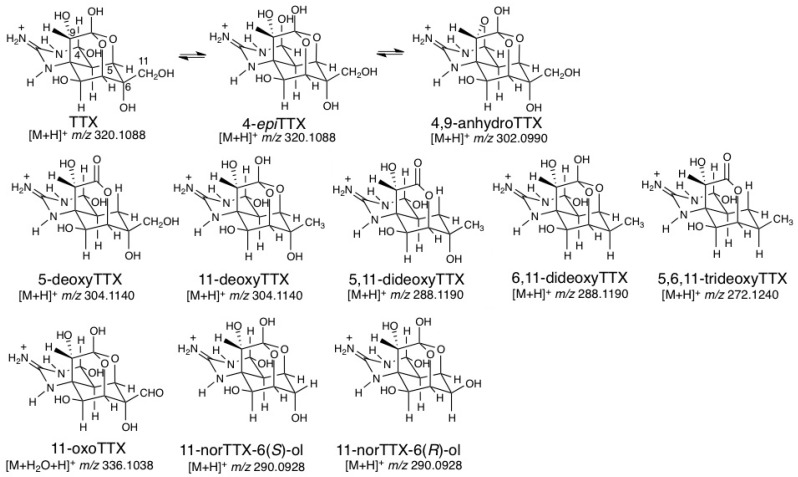
Structures of natural tetrodotoxin (TTX) analogs and the calculated masses for their [M + H]^+^ or [M + H_2_O + H]^+^ [61].

TTX is a non-protein, low molecular weight neurotoxin. It is a heterocyclic compound that consists of a guanidinium moiety connected to an oxygenated backbone that possesses a 2,4-dioxaadamantane structure with six hydroxyl groups [59]. Altogether, 25 naturally occurring analogs of TTX have been detected [60], and many of these have also been shown to have toxicity potential (Figure 5). TTX analogs found in pufferfish, as well as in gastropods and bivalves, can be classified into four groups [61]: (1) analogs chemically equivalent to TTX (4-epiTTX and 4,9-anhydroTTX), (2) deoxy analogs (5-deoxyTTX, 11-deoxyTTX, 6,11-dideoxyTTX, and 5,6,11-trideoxyTTX), (3) 11-CH2OH oxidized analog (11-oxoTTX), and (4) C11 lacking analogs (11-norTTX-6(S)-ol and 11-norTTX-6(R)-ol).

The first detection method used with TTX was the mouse bioassay, a toxic assay based on the toxic effects shown by mice after intraperitoneal injection [62]. However, like with other toxins, these methods have several limitations (low specificity, low accuracy because of individual variability, ethical issues, etc.).

Several methods based on immunological approaches have been used: surface plasmon resonance (SPR) [63,64] and ELISA [65,66,67]. These methodologies are potentially useful for qualitative identification. Nevertheless, at this moment, these methods are not suitable for routine screening due to the lack of cross-reactivity for all TTX analogs, and, as such, may not identify all the analogs of TTX [14].

The best methodology for qualitative identification is the N2a cell assay, as for CTXs, proposed by [41,68,69,70], since TTX blocks the sodium channels, acting as an antagonist of O/V. Cell survival is related to the concentration of TTX in a directly proportional way; the higher the analyte concentration, the more cell survival will be observed.

**Figure 6:**
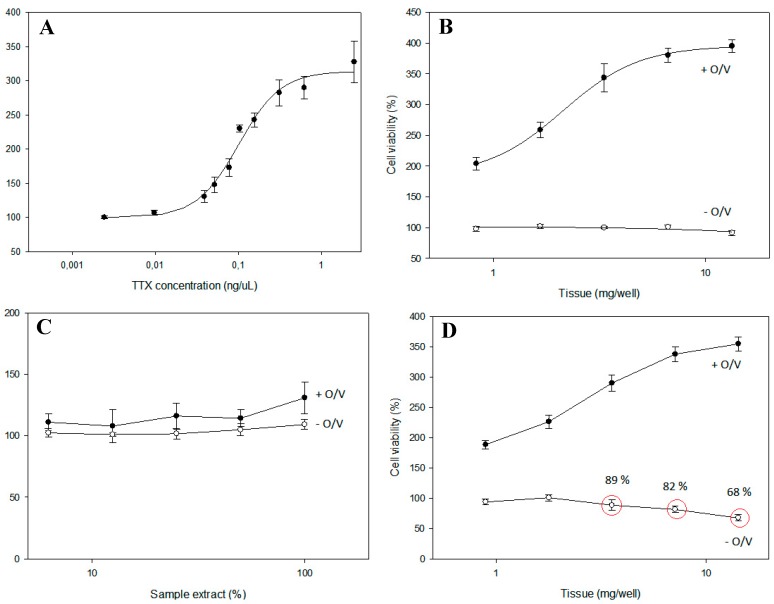
Example of different situations present in the N2a assay. (**A**) TTX standard; (**B**) toxic sample; (**C**) non-toxic sample; (**D**) matrix effect.

This cell assay provides information on the total toxicity related to the sodium channels of the different samples (Figure 6). The main limitations of the cell assay are the lack of specificity; nevertheless, it is a good approach for screening toxicity based on similar mechanisms of action, such as the blocking of sodium channels. Matrix effects must also be considered to avoid false positives.

**Figure 7:**
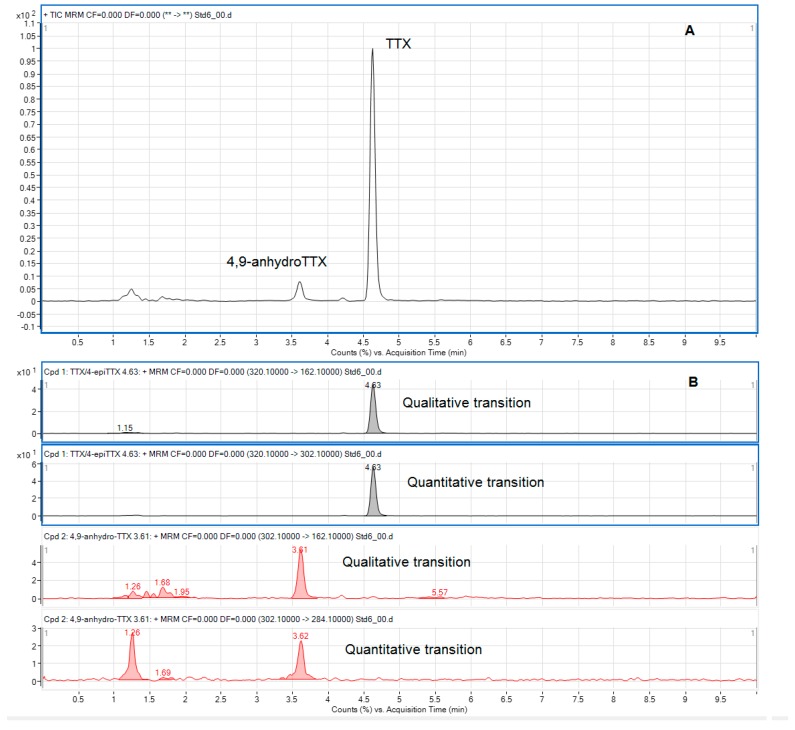
Example of LC-MS/MS (MRM) analysis of a TTX and 4,9-anhidroTTX standard following the EURLMB TTX SOP 2017 [74]. (**A**) Total ion chromatogram; (**B**) Qualitative and quantitative transitions for TTX (in gray) and 4,9-anhidroTTX (in red).

Regarding physicochemical methods, the most widely used are the chromatographic methods (both gas and liquid chromatography) coupled to different detection modes, in particular to mass spectrometry due to its potential for confirmation. Hydrophilic interaction liquid chromatography (HILIC-LC) has been widely recommended for chemical structures like TTXs [25,71,72,73] due to its ability to effectively separate small polar compounds on polar stationary phases. Therefore, this chromatographic approach has been selected as the separation method of choice for these particular compounds while its coupling to mass spectrometry (MS) also plays an important role for confirmation purposes. HILIC–LC-MS/MS is, therefore, the method recommended by the European Union Reference Laboratory for Marine Biotoxins (EURLMB) for the determination of TTXs. The method has been interlaboratory validated [74] and transferred to the EU National Reference Laboratories (NRLs) to help with the evaluation of the occurrence data of TTX in mollusks from their production areas (Figure 7).

## 4. Conclusions

The characterization of the risk of the presence of emerging marine biotoxins in European coastal areas is a priority concern for the EU Health Authorities. Occurrence data are necessary to characterize the risk, and the development of analytical methods for screening and confirmation is strictly required for this purpose. The development of reference materials is still a pending issue that is hampering the advance of both risk evaluation and characterization.

## Figures and Tables

**Table 1:** Main marine biotoxins in seafood, the syndromes, and the legal limits in the EU. Regulation (EC) No. 853/2004; 854/2004; 15/2011, 786/2013.

Syndrome	Reference Compound	Main Toxins in Syndrome	Seafood Affected	EU Limits
**Lipophilic Toxins**				
Diarrhetic Shellfish Poisoning	Okadaic acid	OA, DTX-1,2	shellfish	160 µg OA-eq/kg
-	Yessotoxins	YTX and analogs	shellfish	3.75 mg YTX-eq/kg shellfish
Azaspiracid Poisoning	Azaspiracids	AZA 1–3	shellfish	160 µg AZA1-eq/kg
“Fast-acting toxins”	Cyclic Imines	SPX, Gymnodimines	shellfish	Not regulated
Ciguatera Fish Poisoning	Ciguatoxins	C-CTX, P-CTX, I-CTX, Gambiertoxins, Gambierol	fish	*
Neurotoxic Shellfish Poisoning	Brevetoxin	PbTx-1,2,3,6,7,9,10, Cysteine, glycine metabolites	shellfish	Not regulated
**Hydrophilic Toxins**				
Amnesic Shellfish Poisoning	Domoic acid	Isodomoic and epidomoic acids	shellfish, fish	20 mg DA/kg
Paralytic Shellfish Poisoning	Saxitoxin	Carbamate, N-sulfocarbamoyl, Decarbamoyl	shellfish	800 µg STX-eq 2-HCl/kg
Pufferfish Poisoning	Tetrodotoxin	TTX and analogs	shellfish, fish	**

* Fishery products containing ciguatoxins must not be placed in the market (in accordance with Regulation (EC) No. 853/2004). ** Fishery products derived from the following families must not be placed on the market: Tetraodontidae, Molidae, Diodontidae, and Canthigasteridae. Recommended level proposed by EFSA: 44 µg TTX/kg. OA, Okadaic acid; DTX, dinophysistoxin; YTX, yessotoxin; AZA, azaspiracid; SPX, spirolide; CTX, ciguatoxin; PbTx, brevetoxin; DA, domoic acid; STX, saxitoxin; TTX, tetrodotoxin.
